# 51 Utilizing Indirect Intrapleural Pressure to Guide Mechanical Ventilation in Burn Patients with ARDS

**DOI:** 10.1093/jbcr/iraf019.051

**Published:** 2025-04-01

**Authors:** Amanda Soo Ping Chow, Laura Johnson, Melissa McLawhorn, Lauren Moffatt, Taryn Travis, Jeffrey Shupp, Shawn Tejiram

**Affiliations:** The Burn Center at MedStar Washington Hospital Center; Walter L. Ingram Burn Center at Grady Memorial Hospital; MedStar Health Research Institute; MedStar Health Research Institute; MedStar Washington Hospital Center; MedStar Washington Hospital Center; MedStar Washington Hospital Center

## Abstract

**Introduction:**

Patients with severe burn injuries are at increased risk of developing acute respiratory distress syndrome (ARDS). There is a paucity of literature examining mechanical ventilation strategies such as an open lung approach in burn injured patients with ARDS. Use of esophageal manometry may provide guidance on optimal positive end expiratory pressure (PEEP) required to prevent alveolar collapse while ensuring safe plateau pressures to prevent barotrauma. The aim of this work was to report on a single institution’s use of esophageal manometry in burn injured patients with ARDS to examine outcomes in mortality, oxygenation, and resolution of ARDS.

**Methods:**

Burn injured patients admitted to an ABA verified burn center from January 2017 to May 2024 were retrospectively reviewed for those who required mechanical ventilation for more than 48 hours, developed ARDS by the Berlin definition, and received esophageal manometry to determine optimal PEEP, safe plateau pressures, and airway pressures. Medical records were reviewed for demographics, injury characteristics, ventilator settings, intrapleural pressures obtained by manometry, and arterial partial pressure of oxygen. Oxygenation indices (OI) [(Fraction of inspired oxygen x mean airway pressure) / arterial partial pressure of oxygen] were calculated to compare hypoxic respiratory failure (HRF) severity prior to manometry use, and at 1-, 3- and 5-days following manometry initiation. Friedman’s test was used to compare OI levels among the four time points.

**Results:**

Of 242 patients who required mechanical ventilation during this period, 32 developed ARDS. Overall mortality rate was 30.1%, with 24.6% in the non-ARDS cohort compared to 71.9% in the ARDS cohort (p< 0.0001). Esophageal manometry was utilized in 25 of the 32 patients with ARDS. Of these 25 patients, the median (IQR) TBSA burned was 41% (29-50). Oxygenation indices improved at all subsequent time points after utilization of esophageal manometry, with statistically significant improvements between post manometry days 1 and 5 [OI: 17.7 (8.9-21.4) vs OI: 11.0 (8.6-12.6); p=0.02] and days 3 and 5 [OI: 14.8 (9.3-19.6) vs OI: 11.0 (8.6-12.6); p=0.04]. Despite having median revised Baux scores of 83 (72.1-97.5), 10 out of the 25 (40%) patients with ARDS who utilized manometry had resolution of ARDS.

**Conclusions:**

A strategy incorporating esophageal manometry to determine optimal PEEP and safe plateau pressure can be used to guide ventilator management in burn injured patients with ARDS. Manometry provides an additional tool to help rescue patients who have a high risk of mortality due to the severity of their injury.

**Applicability of Research to Practice:**

Esophageal manometry can be used to provide an individualized approach to mechanical ventilation and improve oxygenation in burn patients with ARDS.

**Funding for the Study:**

N/A

